# Comparative study of National Institute of Health criteria and TNM staging system in predicting the prognosis of gastrointestinal stromal tumours: a retrospective study

**DOI:** 10.3389/fonc.2025.1622777

**Published:** 2025-08-19

**Authors:** Jin-hu Chen, Zhen-rong Yang, Zhi-ming Cai, Tao Lin, Ren Lin, Xin-cheng Su, Rong-bin Kang, Lu Lin, Zai-sheng Ye, Yong-jian Zhou

**Affiliations:** ^1^ Department of Gastric Surgery, Clinical Oncology School of Fujian Medical University, Fujian Cancer Hospital, Fuzhou, China; ^2^ Department of Gastric Surgery, Fujian Medical University Union Hospital, Fuzhou, China

**Keywords:** gastrointestinal stromal tumours, National Institutes of Health (NIH) criteria, TNM staging system, cancer-specific survival, prognostic model

## Abstract

**Background:**

In 2009, the American Joint Commission on Cancer incorporated the gastrointestinal stromal tumours (GISTs) risk classification into the tumour, node, metastasis (TNM) staging system. We aimed to evaluate the prognostic value of the TNM staging system for GISTs by directly comparing it with the modified National Institutes of Health (NIH) criteria.

**Materials and methods:**

We /used data from the Surveillance, Epidemiology, and End Results (SEER) database (2010–2019) to retrospectively analyse patients with gastric and small intestinal/colorectal GISTs. Multivariate Cox regression analysis was performed to identify independent prognostic factors for cancer-specific survival (CSS). To assess the predictive performance of the TNM staging system and the modified NIH criteria, we calculated the area under the receiver operating characteristic curve (AUC), concordance index (C-index), Akaike information criterion (AIC), and Bayesian information criterion (BIC).

**Results:**

Of the 3,034 patients included, 2,106 had gastric GISTs and 928 had small intestinal/colorectal GISTs. Multivariate Cox analysis revealed that TNM stage was an independent prognostic factor for CSS. According to the modified NIH criteria, both the overall and subgroup cohorts exhibited better CSS in the low-risk group than that in the very low-risk group. In contrast, for the TNM staging system, the difference in CSS between stages IIIA and IIIB were not statistically significant (all P>0.05). Notably, only 2 of the 928 patients with small intestinal/colorectal GISTs met the modified NIH criteria for intermediate risk. In the gastric GISTs cohort, the AUC, C-index, AIC, and BIC values for the TNM staging system and the modified NIH criteria were similar. However, in the small intestine and colorectal GISTs cohort, the TNM staging system demonstrated better discriminatory performance with higher AUC and C-index and lower AIC and BIC values compared with the modified NIH criteria.

**Conclusions:**

Regarding prognostic evaluation, the TNM staging system was comparable to the modified NIH criteria for patients with gastric GISTs, but it outperformed the modified NIH criteria in the prediction of outcomes for patients with small intestine and colorectal GISTs.

## 1 Introduction

The previously accepted and widely used risk assessment method for gastrointestinal stromal tumours (GISTs) is the National Institutes of Health (NIH) risk stratification criteria, proposed by Fletcher et al. (1) in 2002. Based on the maximum tumour diameter and mitotic count, the risk of recurrence of GISTs is divided into four grades: very low, low, intermediate, and high. After the NIH criteria were proposed, based on the follow-up data of a large number of GIST cases, Miettinen et al. (2, 3) found that intestinal GISTs were more invasive than gastric GISTs. Therefore, they put forward another risk stratification standard, the Armed Force Institute of Pathology criteria (4). The introduction of tumour location as a parameter improved the accuracy of risk stratification. To address the deficiency of the NIH criteria and improve the complexity of the Armed Force Institute of Pathology standard, in 2008, Joensuu et al. (5) proposed modified NIH criteria. Subsequently, the nomogram (6), contour maps (7), and tumour-grade-metastasis staging systems (8) were applied to the prognostic risk assessment of GISTs. However, these methods lack wide application in clinical practice. With advances in precision oncology, molecular classification based on mutations of genes such as *KIT*, *PDGFRA*, and *SDHB* has emerged as an important prognostic tool in GISTs, whereas rare alterations involving the *NF1*, *BRAF*, or *NTRK* genes may confer resistance to standard tyrosine kinase inhibitors but responsiveness to specific targeted therapies (9, 10). Genomic models such as the MSK–IMPACT-based system further highlight the potential of integrating molecular features into risk stratification (11). However, such models have not yet been incorporated into the NIH or tumour, node, metastasis (TNM) systems and remain primarily research-focused.

Based on the Armed Force Institute of Pathology standard, in 2009, the American Joint Commission on Cancer (AJCC) incorporated the risk classification of GISTs into the TNM staging system for the first time. The updated eighth edition (12) of GIST TNM staging in 2017 was not modified from the seventh edition (13). Previous studies have verified the effectiveness of the TNM staging system, and some studies have suggested that the TNM staging system may be better than the modified NIH criteria in predicting the prognosis of GISTs (14–16). However, despite the formal introduction of the TNM staging system by the AJCC in 2009, it has not been widely adopted in clinical settings for GIST management, with many institutions continuing to rely on the modified NIH criteria. The relative prognostic value and clinical utility of these two systems remain unclear in real-world practice. Therefore, a direct comparison is warranted to provide evidence that may support standardized staging practices across different institutions.

## 2 Materials and methods

### 2.1 Data sources

**Figure 1:**
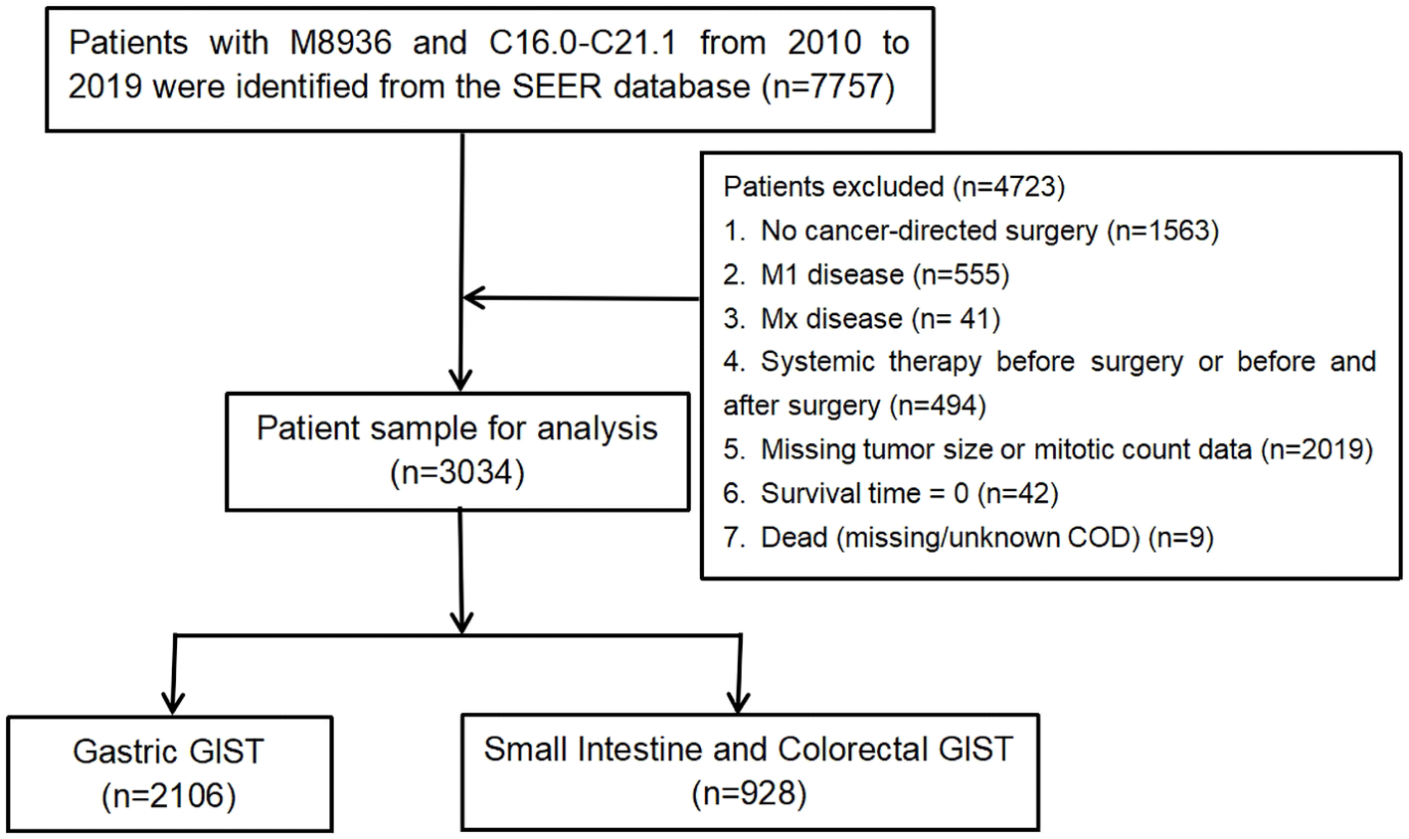
Flowchart of the study selection process. COD, cause of death; GISTs, gastrointestinal stromal tumours.

This study aimed to retrospectively analyse data from the Surveillance, Epidemiology, and End Results (SEER) program database. We used SEER Stat software (SEER*Stat 8.4.1, Incidence-SEER 19 Regs Custom Data with additional treatment fields, Nov 2019 Sub [1973–2019 varying]) to retrieve the data for all patients diagnosed with GISTs (M8936, C16.0–C21.1) between 2010 and 2019. Patients with no cancer-directed surgery (n=1,563), M1 disease indicated as “M1 or Mx” (n=596; Mx cases were excluded because an indeterminate metastatic status might have compromised staging accuracy and introduce systematic bias if included), systemic therapy before surgery or before and after surgery (n=494), and missing tumour size or mitotic count data (n=2,019; handled using listwise deletion), were excluded. Patients with survival times listed as “0” (n=42) or with a SEER cause-specific death classification of “missing/unknown cause of death (COD)” (n=9) were also excluded. Ultimately, a total of 3,034 patients were included, of which 2,106 had gastric GISTs and 928 had small intestine and colorectal GISTs. (**Figure 1**). Because of the AJCC TNM system, gastric GISTs and small intestinal GISTs were classified differently, and colorectal GISTs were classified into the same cohort in this study. Cancer-specific survival (CSS) was chosen over overall or progression-free survival as the primary endpoint because the SEER database provides cause-specific death data but lacks progression information, and overall survival may be confounded by non-cancer-related deaths in older patients. CSS was calculated from the date of GIST diagnosis to either cancer-related death or the most recent follow-up. As the SEER database provides de-identified, publicly available data, which does not involve any risk to personal privacy, institutional review board approval and written consent from participants were not required.

### 2.2 TNM staging system

The 8th edition (12) of the AJCC TNM staging system (2017) was applied to classify patients with GISTs. It incorporates tumour size (T), lymph node involvement (N), distant metastasis (M), and mitotic activity to stratify patients into prognostic stage groups. Tumour size categories were as follows: T1 (≤2 cm), T2 (>2 cm and ≤5 cm), T3 (>5 cm and ≤10 cm), and T4 (>10 cm). Nodal status was defined as N0 (no regional lymph node metastasis or unknown status) or N1 (regional lymph node metastasis). Distant metastasis was defined as M0 (absent) or M1 (present). Mitotic rate was graded as low (≤5 mitoses per 5 mm^2^ or per 50 high-power fields [HPFs]) or high (>5 mitoses per 5 mm^2^ or per 50 HPFs).

Stage groupings were as follows:

Gastric GISTs:

- Stage IA: T1 or T2, N0, M0, and low mitotic rate;
- Stage IB: T3, N0, M0, and low mitotic rate;
- Stage II: T1 or T2, N0, M0, and high mitotic rate; or T4, N0, M0, and low mitotic rate;
- Stage IIIA: T3, N0, M0, and high mitotic rate;
- Stage IIIB: T4, N0, M0, and high mitotic rate;
- Stage IV: Any T, N1 or M1, and any mitotic rate.

Small intestine and colorectal GISTs:

- Stage I: T1 or T2, N0, M0, and low mitotic rate;
- Stage II: T3, N0, M0, and low mitotic rate;
- Stage IIIA: T1, N0, M0, and high mitotic rate; or T4, N0, M0, and low mitotic rate;
- Stage IIIB: T2, T3, or T4, N0, M0, and high mitotic rate;
- Stage IV: Any T, N1 or M1, and any mitotic rate.

The gastric staging system also applies to omental GISTs, while small intestinal staging also applies to oesophageal, colorectal, mesenteric, and peritoneal GISTs.

### 2.3 Statistical analysis

Statistical analyses were performed using IBM SPSS Statistics version 25.0 (IBM Corp., Armonk, NY, USA) and R software version 4.4.2. Descriptive statistics for sex, age, race, primary site, tumour size, mitotic count, N stage, modified NIH criteria, and TNM stage were summarized as frequencies and percentages (%). Univariate survival analysis was conducted using the Kaplan–Meier method and log-rank tests. Multivariate analysis was performed using the Cox proportional-hazards regression model to identify independent risk factors associated with CSS. The proportional-hazards assumption for the Cox models was tested using Schoenfeld residuals and found to be satisfied. Kaplan–Meier analysis was also used to estimate 5-year CSS rates across different subgroups defined according to the TNM staging system and the modified NIH criteria in the three cohorts, with pairwise comparisons between adjacent subgroups evaluated via log-rank tests. Univariate Cox regression analysis was used to calculate hazard ratios (HRs) for each subgroup within the TNM staging system and modified NIH criteria. The prognostic performance of the TNM staging system and the modified NIH criteria was compared using the area under the receiver operating characteristic curve (AUC), concordance index (C-index), Akaike information criterion, and Bayesian information criterion. The C-index and its 95% confidence intervals (CIs) were calculated using 1,000 bootstrap resamples. This bootstrap procedure served as internal validation to assess the robustness of model discrimination. Differences in C-index between models were tested using the DeLong method. A P-value < 0.05 was considered statistically significant.

## 3 Results

### 3.1 Patient characteristics

We retrospectively analysed the data of 3,034 patients with GISTs who had not undergone preoperative neoadjuvant therapy, according to the SEER database. Of these, 2,106 patients had gastric GISTs and 928 had small intestine and colorectal GISTs. All the patients underwent tumour-related surgery and had complete data that could be used for analysis with the modified NIH criteria and TNM staging system. Univariate analysis showed that age, primary site, tumour size, mitotic count, N stage, NIH criteria, and TNM stage were prognostic factors for patients with GISTs. The proportion of patients in each subgroup, and 5-year CSS rates are shown in **Table 1**. This study protocol was approved by the Ethics Committee of Fujian Cancer Hospital (Fuzhou, China). The SEER database is publicly available and provides data of de-identified cases. As the analysis used anonymous clinical data, the requirement of written informed consent was waived.

**Table 1:** Demographic and clinicopathological characteristics of the 3,034 patients with GISTs.

Variables	Patients (%)	Univariate analysis	
	N=3,034	5-year CSS (%)	P value
Gender			0.054
Male	1,472 (48.5)	92.7	
Female	1,562 (51.5)	94.3	
Age, y, Median (Min–Max)	64 (10–95)		<0.001
≤ 60	1,192 (39.3)	96.0	
>60	1,842 (60.7)	91.8	
Race			0.070
White	2,059 (67.9)	92.9	
Black	538 (17.7)	91.9	
Other	437 (14.4)	94.0	
Primary site			0.009
Stomach	2,106 (69.4)	94.0	
Small intestine	881 (29.0)	92.1	
Colo-rectum	47 (1.5)	97.8	
Tumour size (cm)			<0.001
≤2	500 (16.5)	95.5	
>2, ≤5	1,171 (38.6)	97.2	
>5, ≤10	918 (30.3)	91.7	
>10	445 (14.7)	86.1	
Mitotic count			<0.001
≤5	2,451 (80.8)	95.6	
>5, ≤10	237 (7.8)	90.2	
>10	346 (11.4)	81.5	
N stage			<0.001
N0	2,996 (98.7)	93.6	
N1	38 (1.3)	85.7	
NIH criteria			<0.001
Very low risk	486 (16.0)	95.4	
Low risk	1,001 (33.0)	97.9	
Intermediate risk	490 (16.2)	95.6	
High risk	1,057 (34.8)	87.8	
TNM stage			<0.001
I	1,893 (62.4)	96.6	
II	564 (18.6)	94.2	
IIIA	257 (8.5)	85.7	
IIIB	282 (9.3)	81.1	
IV*	38 (1.3)	85.7	

*Stage IV in this table includes only N1 cases and not M1 cases. CSS, cancer-specific survival; NIH, National Institutes of Health; TNM, tumour, node, metastasis.

### 3.2 Independent prognostic factors of GISTs

**Figure 2:**
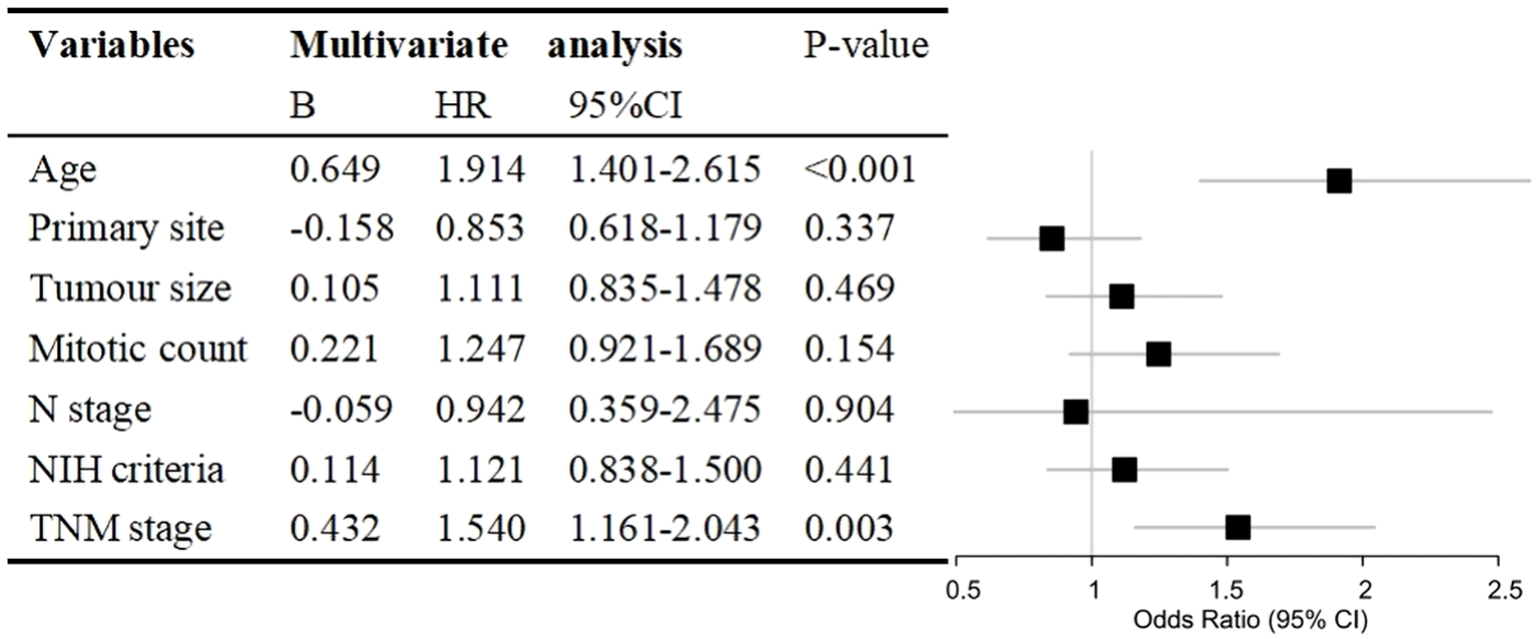
Hazard ratio (HR) with 95% confidence interval (CI) estimated via multivariate Cox regression analysis of prognostic factors for cancer-specific survival in patients with gastrointestinal stromal tumours. NIH, National Institutes of Health; TNM, tumour, node, metastasis.

Variables with P<0.05 in the univariate analyses were included in the multivariate Cox regression analysis. We discovered that age (HR, 1.914; 95% CI, 1.401–2.615; P<0.001) and TNM stage (HR, 1.540; 95% CI, 1.161–2.043; P=0.003) were independent prognostic factors for CSS in patients with GISTs. Primary site, tumor size, mitotic count, N stage, and NIH criteria were not independent prognostic factors (**Figure 2**). The lack of significance of tumor size and mitotic count may be explained by their incorporation into the TNM staging system, which likely led to collinearity and loss of independent predictive power in the multivariate model.

### 3.3 Comparison of the two prognostic classification systems in the entire cohort

**Figure 3:**
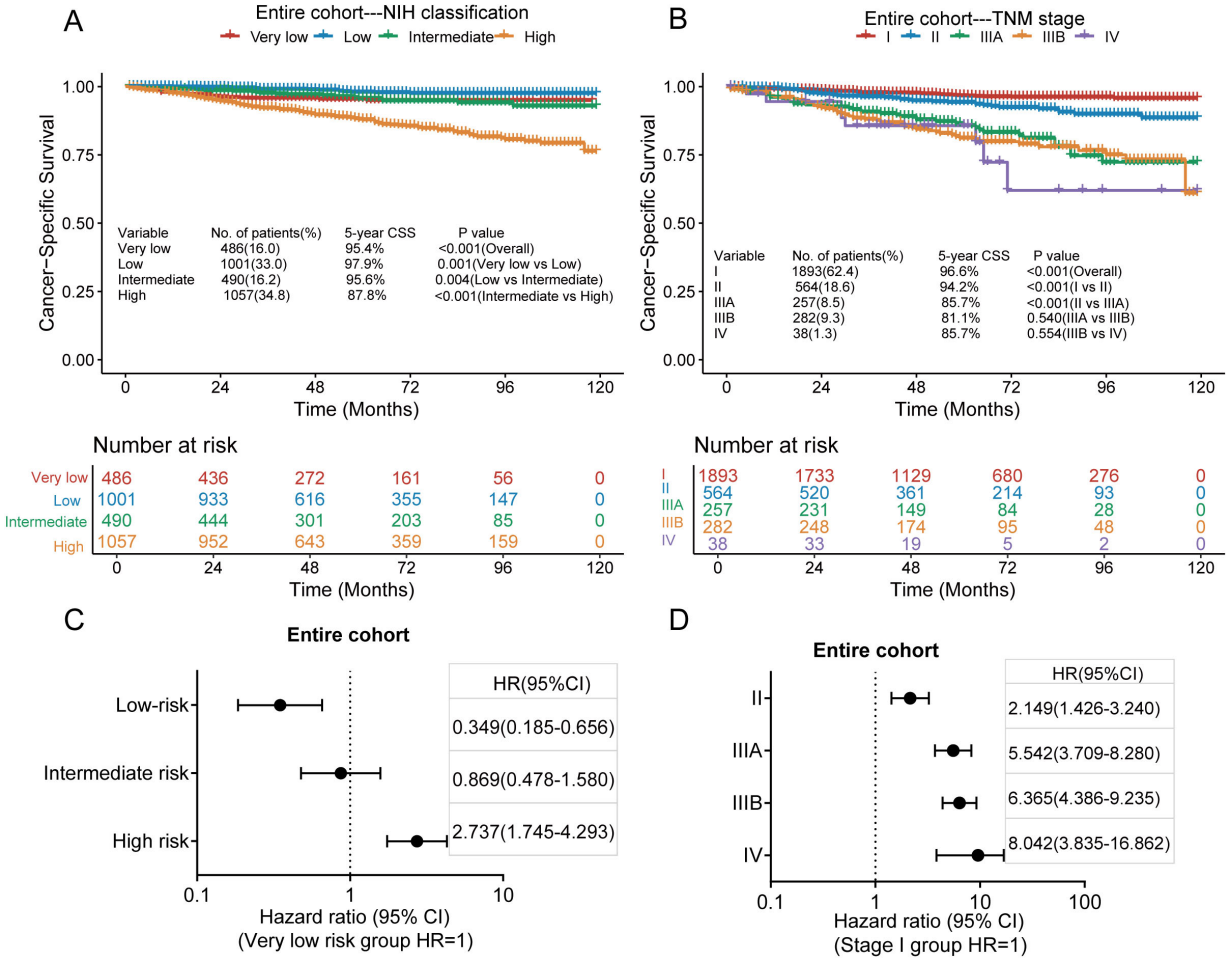
Kaplan–Meier survival plots of cumulative cancer-specific survival (CSS) using the modified National Institutes of Health (NIH) criteria **(A)** and the tumour, node, metastasis (TNM) staging system **(B)**. The hazard ratio (HR) obtained from the Cox regression model for the different staging systems in the entire cohort via the modified NIH criteria **(C)** and the TNM staging system **(D)**. CI, confidence interval.

The median follow-up duration for all patients was 58 months (range, 1–119 months). Of the 3034 patients included in our analysis, 198 died of GISTs. The 3- and 5-year CSS rates were 95.8% and 93.5%, respectively. Kaplan–Meier survival analysis showed significant differences in cancer-specific survival rates among very low-, low-, intermediate-, and high-risk groups in the modified NIH criteria (**Figure 3A**). The 5-year CSS rates of these risk groups were 95.4%, 97.9%, 95.6%, and 87.8%, respectively. Significant differences were also seen in CSS between stages I, II, and IIIA in the TNM staging system. However, no significant differences were found between stages IIIA, IIIB, and IV (**Figure 3B**). The 5-year CSS rates for stages I, II, IIIA, IIIB, and IV were 96.6%, 94.2%, 85.7%, 81.1%, and 85.7%, respectively. According to the modified NIH criteria, the HRs of low-risk, intermediate-risk, and high-risk groups were 0.349 (95% CI, 0.185–0.656), 0.869 (95% CI, 0.478–1.580), and 2.737 (95% CI, 1.745–4.293), respectively, compared with the very low-risk group (**Figure 3C**). According to the TNM staging system, the HRs for stages II, IIIA, IIIB, and IV were 2.149 (95% CI, 1.426–3.240), 5.542 (95% CI, 3.709–8.280), 6.365 (95% CI, 4.386–9.235), and 8.042 (95% CI, 3.835–16.862), respectively, compared with stage I (**Figure 3D**).

### 3.4 Comparison of the two prognostic classification systems in the gastric GISTs cohort

**Figure 4:**
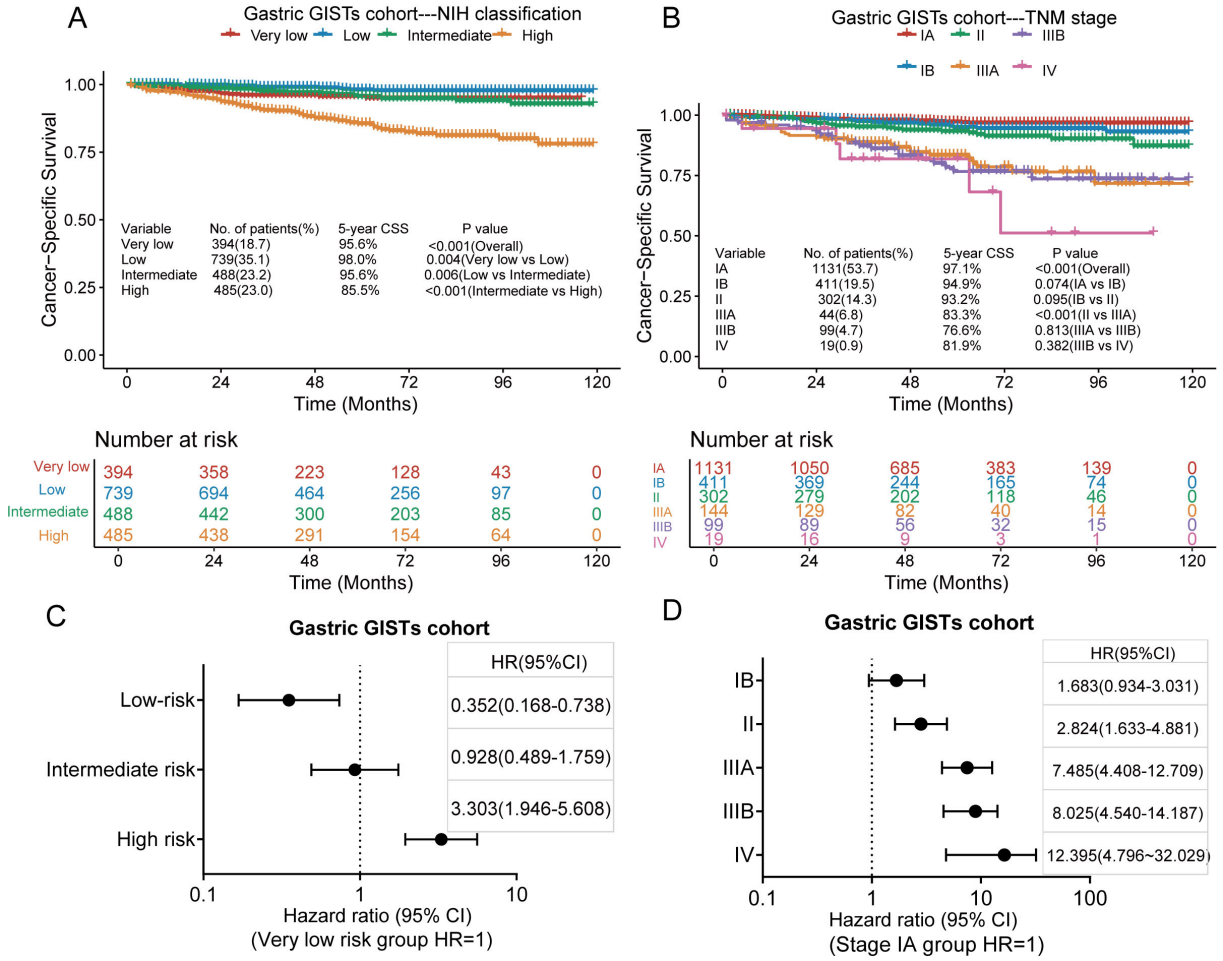
Kaplan–Meier survival plots of cumulative cancer-specific survival (CSS) rates in the gastric GISTs cohort using the modified National Institutes of Health (NIH) criteria **(A)** and the tumour, node, metastasis (TNM) staging system **(B)**. The hazard ratio (HR) obtained from the Cox regression model for the different staging systems in the gastric GISTs cohort using the modified NIH criteria **(C)** and the TNM staging system **(D)**. CI, confidence interval.

Of the 2,106 patients with gastric GISTs, 121 died of GISTs, and the 3-and 5-year CSS rates were 96.1% and 94.0%, respectively. Kaplan–Meier survival analysis showed a significant difference in CSS between the very low-risk, low-risk, intermediate-risk, and high-risk groups in the modified NIH criteria (**Figure 4A**); however, no significant difference was found in CSS between the very low-risk and intermediate-risk groups. The 5-year CSS rates for very low, low, intermediate, and high-risk groups were 95.6%, 98.0%, 95.6%, and 85.5%, respectively. Kaplan–Meier survival analysis showed a significant difference in CSS between stages II and IIIA in the TNM staging system, but no difference was observed between any other adjacent pair of stages (**Figure 4B**). The 5-year CSS rates for stages IA, IB, II, IIIA, IIIB, and IV were 97.1%, 94.9%, 93.2%, 83.3%, 76.6%, and 81.9%, respectively. According to the modified NIH criteria, the HRs of low-risk, intermediate-risk, and high-risk groups were 0.352 (95% CI, 0.168–0.738), 0.928 (95% CI, 0.489–1.759), and 3.303 (95% CI, 1.946–5.608), respectively, compared with the very low-risk group (**Figure 4C**). According to the TNM staging system, the HRs for stages IB, II, IIIA, IIIB, and IV were 1.683 (95% CI, 0.934–3.031), 2.824 (95% CI, 1.633–4.881), 7.485 (95% CI, 4.408–12.709), 8.025 (95% CI, 4.540–14.187), and 12.395 (95% CI, 4.796–32.029), respectively, compared with stage IA (**Figure 4D**).

### 3.5 Comparison of the two prognostic classification systems in the small intestine and colorectal GISTs cohort

**Figure 5:**
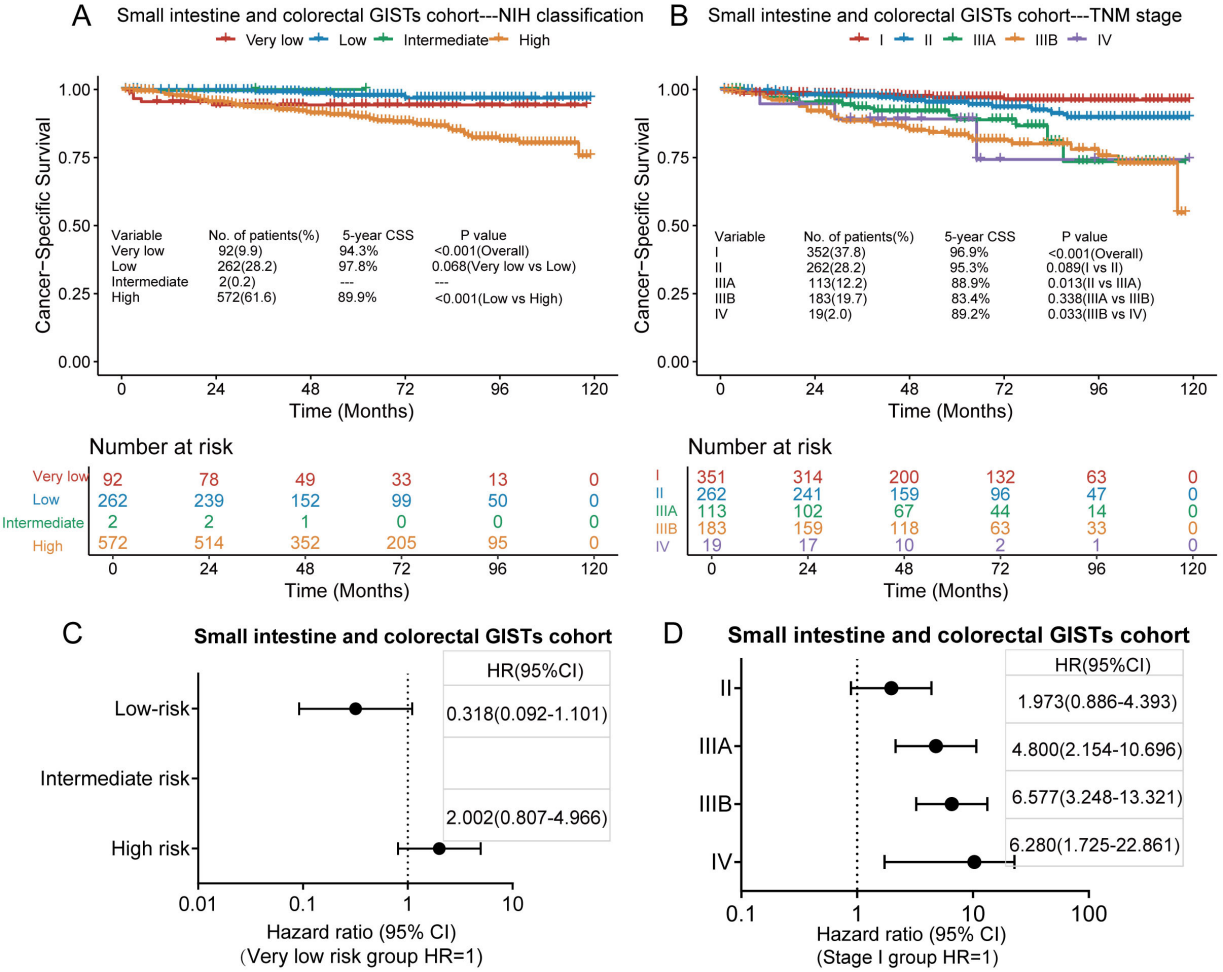
Kaplan–Meier survival plots of cancer-specific survival (CSS) for the small intestine and colorectal GISTs cohort using the modified National Institutes of Health (NIH) criteria **(A)** and the tumour, node, metastasis (TNM) staging system **(B)**. The hazard ratio (HR) obtained from the Cox regression model for the different staging systems in the small intestine and colorectal GISTs cohort using the modified NIH criteria **(C)** and the TNM staging system **(D)**. CI, confidence interval.

Of the 928 patients with small intestine and colorectal GISTs, 77 died of GISTs, and the 3-and 5-year CSS rates were 95.1% and 92.4%, respectively. In the small intestine and colorectal GISTs cohort, only two cases were classified as intermediate risk based on the modified NIH standard analysis. Kaplan–Meier survival analysis showed a significant difference in CSS only between the low-risk and high-risk groups in the modified NIH criteria (**Figure 5A**), while there was no significant difference in CSS between the very low-risk and low-risk groups. The 5-year CSS rates in the very low-, low-, and high-risk groups were 94.3%, 97.8%, and 89.9%, respectively. Kaplan–Meier survival analysis showed that, in the TNM staging system, significant differences in CSS occurred between stages II and IIIA and stages IIIB and IV but not between the other stages (**Figure 5B**). The 5-year CSS rates for stages I, II, IIIA, IIIB, and IV were 96.9%, 95.3%, 88.9%, 83.4%, and 89.2%, respectively. According to the modified NIH criteria, the HRs of the low-risk and high-risk groups compared to the very low-risk group were 0.318 (95% CI, 0.092–1.101) and 2.002, (95% CI, 0.807–4.966), respectively (**Figure 5C**). According to the TNM staging system, the HRs for stages II, IIIA, IIIB, and IV to stage I were 1.973 (95% CI, 0.886–4.393), 4.800 (95% CI, 2.154–10.696), 6.577 (95% CI, 3.248–13.321), and 6.280 (95% CI, 1.725–22.861), respectively (**Figure 5D**).

### 3.6 Comparison of the two prognostic classification systems for CSS

**Figure 6:**
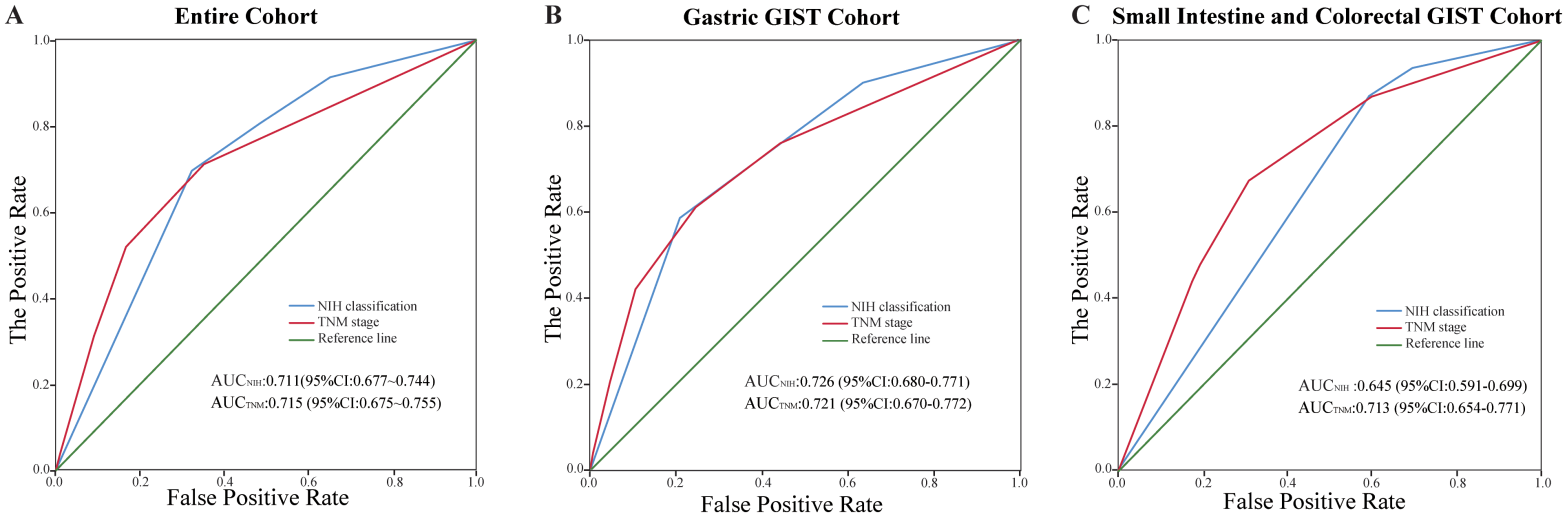
Comparison of the performance of the modified National Institutes of Health (NIH) criteria and the tumour, node, metastasis (TNM) staging system. **(A)** Entire cohort, **(B)** gastric gastrointestinal stromal tumours (GISTs) cohort, and **(C)** small intestine and colorectal GISTs cohort. AUC, area under the receiver operating characteristic curve; CI, confidence interval.

In the entire GISTs cohort, the prognostic value of the TNM staging system (AUC, 0.715; 95% CI, 0.675–0.755) was similar to that of the modified NIH criteria (AUC, 0.711; 95% CI, 0.677–0.744) (**Figure 6A**). In the gastric GISTs cohort, the TNM staging system (AUC, 0.721; 95% CI, 0.670–0.772) was also comparable to the modified NIH criteria (AUC, 0.726; 95% CI, 0.680–0.771) in terms of prognostic value (**Figure 6B**). In the small intestine and colorectal GISTs cohort, the prognostic discriminatory ability of the TNM staging system (AUC, 0.713; 95% CI, 0.654–0.771) was better than that of the modified NIH criteria (AUC, 0.645; 95% CI, 0.591–0.699) (**Figure 6C**). In all cohorts, the C-index of the NIH criteria (all GISTs: 0.70 [95% CI, 0.67–0.73]; gastric GISTs: 0.72 [95% CI, 0.68–0.76]) was similar to that of the TNM stage (all GISTs: 0.69 [95% CI, 0.65–0.73]; gastric GISTs: 0.69 [95% CI, 0.64–0.74]), while the C-index of the NIH criteria (small intestine and colorectal GISTs: 0.62 [95% CI, 0.58–0.66]) was significantly lower than that of the TNM stage (small intestine and colorectal GISTs: 0.69 [95% CI, 0.62–0.74]) (P<0.001). In the small intestine and colorectal GIST cohort, both the Akaike information criterion and Bayesian information criterion of the TNM stage were lower than those of the NIH criteria (**Table 2**).

**Table 2:** C-Index, AIC, and BIC for NIH criteria and TNM stage by cohort.

Variable	NIH criteria	TNM stage	P value
All GISTs			
C-index (95% CI)	0.70 (0.67 to 0.73)	0.69 (0.65 to 0.73)	0.686
AIC	2908.45	2897.85	
BIC	2918.31	2911.01	
Gastric GISTs			
C-index (95% CI)	0.72 (0.68 to 0.76)	0.69 (0.64 to 0.74)	0.129
AIC	1692.64	1695.56	
BIC	1701.03	1706.74	
Small intestine and colorectal GISTs			
C-index (95% CI)	0.62 (0.58 to 0.66)	0.69 (0.62 to 0.74)	<0.001
AIC	956.01	944.14	
BIC	963.04	953.51	

NIH, National Institutes of Health; TNM, tumour, node, metastasis; GISTs, gastrointestinal stromal tumours; C-index, concordance index; CI, confidence interval; AIC, Akaike information criterion; BIC, Bayesian information criterion.

## 4 Discussion

This study aimed to determine the value of the TNM staging system in evaluating the prognosis of GISTs, by comparing it with the most widely used modified NIH criteria. We found that the prognosis predicted by the TNM staging system was comparable to that of the modified NIH criteria in the entire GISTs cohort and in the gastric GISTs cohort. However, in the small intestine and colorectal GISTs cohort, the TNM staging system was significantly better than the modified NIH criteria in predicting prognosis. Therefore, we believe that in assessing the prognosis of GISTs, the TNM staging system is comparable to the modified NIH criteria for gastric GISTs and superior to the modified NIH criteria for small intestine and colorectal GISTs.

Further, we found that both the modified NIH criteria and the TNM staging system have some limitations in predicting the risk of GISTs. According to the modified NIH criteria, the results in the entire GISTs cohort and subgroups showed that the cancer-specific survival rate in the low-risk group was better than that in the very low-risk group. Cox analysis also showed that the risk of death in the low-risk group was lower than that in the very low-risk group. This finding appears counterintuitive and may be partly due to the limited granularity of early-stage risk stratification under the NIH system. However, unmeasured confounders might also have contributed to this pattern. For example, the SEER database does not contain important clinical information such as surgical margin status, tumour rupture, or adjuvant therapy use. Additionally, potential differences in patient age, comorbidities, or treatment quality between groups might have biased survival outcomes. Therefore, this observation should be interpreted with caution. Zhao et al. (17) found that according to the NIH criteria, the 3-year disease-specific survival rates of very low and low risks were 99.5% and 99.4% respectively, and the difference was not statistically significant (p= 0.932). Oweira et al. (16) also found that pairwise comparisons among different NIH scale categories were non-significant except for intermediate risk compared with high-risk, and high-risk compared with metastatic disease. The modified NIH criteria for very low and low risks were a tumour size of ≤ 2 cm and tumour size of 2.1–5 cm, respectively, as well as a mitotic index of ≤ 5 per 50 HPFs. The tumour size corresponds to that of T1 and T2 in the TNM staging system. Based on a mitotic index of ≤ 5 per 50 HPFs, the TNM staging system is classified as stage I, which may be more feasible than the modified NIH criteria. Therefore, we believe that stratification of patients with early GISTs according to the modified NIH criteria would not be accurate and is unnecessary to use in predicting prognosis, because follow-up is recommended for both low-risk and very low-risk GISTs.

In the entire GISTs cohort and in the subgroups, for the TNM staging system, Cox analysis showed that the risk of death increased with increased stage. However, for the modified NIH criteria, only in the high-risk group was the risk of death higher than that for the very low-risk group. Therefore, the TNM staging system appears more useful. However, we also found that in the entire GISTs cohort and in the subgroups, Kaplan–Meier analysis showed no significant difference in cancer-specific survival between stages IIIA and IIIB. Therefore, we believe that the stratification of relatively advanced cases according to the TNM staging system may be inadequate. Considering that the TNM staging system only divides the mitotic index into ≤ 5 and > 5 per 50 HPFs, the ability of the TNM staging system to stratify the prognosis of patients with GISTs with a high mitotic index may decrease.

In the small intestine and colorectal GISTs cohort, we found that according to the modified NIH criteria, only two cases met the intermediate risk classification (tumour size ≤2 cm, mitotic index 6–10 per 50 HPFs), indicating significant stratification imbalance. This finding highlights a limitation of the NIH system for non-gastric GISTs, where its granularity may be insufficient for clinical decision-making. For instance, if the mitotic index is 4/50 HPFs, a tumour 5.0 cm in diameter is categorized as low risk, while one 5.1 cm in diameter is deemed high risk, leading to vastly different treatment recommendations—surveillance versus ≥3 years of adjuvant imatinib—despite no meaningful clinical distinction. This abrupt treatment divergence caused by a minor size difference suggests that the NIH criteria may lack clinical practicality in this subgroup. Furthermore, as the NIH classification was originally derived from gastric GISTs data, its applicability to small intestine and colorectal GISTs is questionable. Prior studies by Miettinen et al. (3) and Joensuu (5) have shown that intestinal GISTs behave more aggressively than gastric GISTs, underscoring the need for site-specific risk models. In contrast, Kaplan–Meier and Cox regression analyses in our study demonstrated that the TNM staging system provided more consistent and clinically aligned prognostic stratification for small intestine and colorectal GISTs. Taken together, these findings support the view that the modified NIH criteria may not be suitable for risk assessment in non-gastric GISTs and highlight the potential of the TNM system as a more robust alternative.

Importantly, GISTs have entered the molecular era. Molecular testing can identify driver mutations in approximately 99% of patients (9). Numerous studies have confirmed that genotype is an independent prognostic factor and also impacts the response to tyrosine kinase inhibitors (18–20). However, neither the NIH nor TNM systems incorporate genetic data. While these models provide basic clinical stratification, they do not reflect the precision oncology paradigm. Indeed, both the National Comprehensive Cancer Network and the updated 2022 European Society for Medical Oncology guidelines recommend avapritinib for neoadjuvant treatment of GISTs with *PDGFRA* exon 18 mutations (including the imatinib-resistant p.D842V variant), NTRK-targeted therapy for *NTRK* fusion-positive GISTs, and BRAF-targeted agents for *BRAF*-mutant GISTs (21, 22). Therefore, genotypes should urgently be incorporated into GISTs prognostic models. Recently, researchers from Memorial Sloan Kettering Cancer Center developed a genomic risk stratification model based on MSK-IMPACT data (11), proposing molecular-based classifications tailored according to the primary tumour site. For example, gastric GISTs with chr1p loss or *SDHB* deletion are deemed high risk, while those with *KIT* exon 11 deletion or chr14q loss are considered moderate risk. For small bowel GISTs, alterations in *MAX*/*MGA*/*MYC*, *CDKN2A*, or *RB1* confer a high risk, while chr1p deletion or chr5q amplification indicate an intermediate risk. This innovative classification scheme may complement traditional clinicopathologic systems and should be validated in prospective or independent cohorts.

This study has several limitations. First, owing to its retrospective nature and use of the SEER database, key clinical data such as tumour rupture, surgical margin status, and adjuvant imatinib use were unavailable. These factors are known to affect prognosis and might have introduced biases, particularly in evaluating the NIH criteria. Second, the SEER database lacks molecular data, including those on *KIT* and *PDGFRA* mutations, limiting the applicability of our findings in precision oncology. Third, certain subgroups, such as the NIH intermediate-risk category for small intestinal and colorectal GISTs, were small, which might have affected the stability of survival estimates. Future studies with larger or multicentre cohorts are needed to validate these findings.

In conclusion, in terms of prognostic evaluation, the TNM staging system is similar to the modified NIH criteria for patients with gastric GISTs, whereas the TNM staging system is superior to the modified NIH criteria for patients with intestinal and colorectal GISTs. Furthermore, patients with small intestine and colorectal GISTs were rarely classified as intermediate risk according to the modified NIH criteria, suggesting limited stratification granularity and clinical applicability in this subgroup. These findings suggest that using the TNM system in non-gastric GISTs may offer more rational guidance for treatment decisions, particularly in avoiding overtreatment and providing more tailored recommendations regarding adjuvant therapy and follow-up intensity.

## Data Availability

The original contributions presented in the study are included in the article/supplementary material. Further inquiries can be directed to the corresponding authors.
